# From plan to delivery: Machine learning based positional accuracy prediction of multi‐leaf collimator and estimation of delivery effect in volumetric modulated arc therapy

**DOI:** 10.1002/acm2.14437

**Published:** 2024-06-20

**Authors:** Minmin Qiu, Jiajian Zhong, Zhenhua Xiao, Yongjin Deng

**Affiliations:** ^1^ Department of Radiation Oncology The First Affiliated Hospital of Sun Yat‐Sen University Guangzhou China

**Keywords:** dynalog, ensemble learning, multi‐leaf collimator, random forest, volumetric modulated arc therapy

## Abstract

**Purpose:**

The positional accuracy of MLC is an important element in establishing the exact dosimetry in VMAT. We comprehensively analyzed factors that may affect MLC positional accuracy in VMAT, and constructed a model to predict MLC positional deviation and estimate planning delivery quality according to the VMAT plans before delivery.

**Methods:**

A total of 744 “dynalog” files for 23 VMAT plans were extracted randomly from treatment database. Multi‐correlation was used to analyzed the potential influences on MLC positional accuracy, including the spatial characteristics and temporal variability of VMAT fluence, and the mechanical wear parameters of MLC. We developed a model to forecast the accuracy of MLC moving position utilizing the random forest (RF) ensemble learning method. Spearman correlation was used to further investigate the associations between MLC positional deviation and dosage deviations as well as gamma passing rates.

**Results:**

The MLC positional deviation and effective impact factors show a strong multi‐correlation (R = 0.701, *p*‐value < 0.05). This leads to the development of a highly accurate prediction model with average variables explained of 95.03% and average MSE of 0.059 in the 5‐fold cross‐validation, and MSE of 0.074 for the test data was obtained. The absolute dose deviations caused by MLC positional deviation ranging from 12.948 to 210.235 cGy, while the relative volume deviation remained small at 0.470%–5.161%. The average MLC positional deviation correlated substantially with gamma passing rates (with correlation coefficient of −0.506 to −0.720 and *p*‐value < 0.05) but marginally with dosage deviations (with correlation coefficient < 0.498 and *p*‐value > 0.05).

**Conclusions:**

The RF predictive model provides a prior tool for VMAT quality assurance.

## 1 INTRODUCTION

In radiotherapy, volumetric modulated arc therapy (VMAT) requires rapidly delivering variable photon beams whose intensity is modulated by the motion of multi‐leaf collimator (MLC), and enables simultaneous modulations of other mechanical features such as gantry rotation speed and dose‐rate.^1^, ^2^ Due to the choreographed nature of VMAT, many potential sources of error arise.^3^ Quality assurance (QA) is crucial for the precise of dosimetry and the accuracy of MLC position.^4^

Numerous studies that examined the factors influencing MLC position accuracy have been published. For instance, linear relation between the leaf velocity and accuracy have been reported.^5^, ^6^, ^7^ Based on extracting information from the running log files such as leaf position, dose fraction, leaf velocity, leaf moving status and others, prediction of the deviations in individual leaf was also proposed.^8^ However, this information is inaccessible until the delivery of treatment plan completed, and they cannot provide any directory information about how the planning result will influence MLC deviations. Carlson et al.^3^ made efforts to grab leaves positions from VMAT plans to predict errors before implementation. Deviation of MLC position was used as output of the models, and predictive leaf motion parameters such as leaf position, velocity, moving direction and others for the models were calculated from the plan files, which were still not the information that can be obtained intuitively from dose distribution as viewed.

In fact, the control point (CP) sequence is generated by leaf sequencing algorithm (LSA) based on the optimized deliverable fluence. LSA takes various limitations of delivery system into consideration, including maximum allowable MLC leaf velocity (MLV), dose rate (DR), MLC leakage and leaf gap.^9^ When these mechanical limits are assumed to be constant, MLC information will inevitably have correlation with fluence. Calculation based on fluence analysis methods, such as modulation indices, have proposed for verifying the accuracy of IMRT or VMAT plan delivery.^10^, ^11^, ^12^ Textural features of fluence were also calculated to quantify modulation degree.^13^, ^14^ Fluence, however, only reflects the spatial characteristics without considering the temporal variability of CP. When the adjacent CPs switch, the larger difference in shapes, the greater velocity and acceleration of MLC leaves required. Therefore, these methods are insufficient and unable to retrieve complete information.

In addition, we frequently ignore the mechanical wear of MLC as an issue. Each leaf movement unit of MLC comprises a driving motor connected to the screw and nut.^15^ To meet the needs of clinical treatment, these movement units require prolonged back and forth motion, resulting in aging and wear of the motors, nuts and screws. Furthermore, due to accumulation of dust between leaves and grooves, the friction force will increase. These mechanical changes will inevitably affect the moving of MLC, which is a negative effect that cannot be ignored on the positional accuracy. However, there are less studies involved on this field up to now.

In this study, an MLC positional accuracy prediction model based on machine learning was developed based on the field shape and dose distribution information of VMAT plans before delivery. Spatial characteristics and temporal variability of fluence were calculated in the method. We comprehensively analyzed factors that may affect positional accuracy, especially the mechanical wear of MLC which was proposed for the first time. To fit the prediction model, all of the effective factors and MLC positional deviation were utilized. In addition, correlations between positional deviation, dose deviations and gamma passing rates were analyzed in order to estimate the impact of MLC positional accuracy on planning delivery quality, and to prove that our study can provide a prior tool for the accurate quality assurance. Additionally, this study is also a supplement to the lack of dosage study for HD120 MLC with quarter leaf.

## 2 METHODS

### 2.1 Patient data

From a prior treatment database, a total of 23 VMAT plans for Nasopharyngeal cancer (NPC) were retrospectively and arbitrarily retrieved. These plans were made using Eclipse TPS (version 13.5, Varian Medical Systems, Palo Alto, CA), which includes an anisotropic analytic algorithm (AAA, version 13.5, Varian Medical Systems, Palo Alto, CA) for dose calculation and a progressive resolution optimizer (PRO, version 13.5, Varian Medical Systems, Palo Alto, CA) for fluence optimization. Prescription doses of 60–66 Gy, 54 Gy, 60 and 68.1 Gy in 30 fractions were delivered, respectively, to the lymph nodes, planning clinical target volumes 2 (PCTV2, which includes the areas of the skull base, nasal cavity, maxillary sinus, posterior group of sieve sinuses, lower pterygoid sinus, parapharyngeal space, slopes, and lymphatic drainage zones that may be invaded by the tumor), planning clinical target volumes 1 (PCTV1, which is the potential invasion area around the tumor), and planning gross tumor volume (PGTV, which is the area of diagnostic visualization of tumors and their invasion) for each of the plans. The NovalisTX Linac, which is outfitted with a millennium 120HD MLC (Varian Medical Systems, Palo Alto, CA), was used to provide the delivery of these plans with 6 MV photon beams in two full arcs. This MLC system contains 60 leaf pairs overall in each of its two banks (A and B). The 32 quarter‐leaf pairs are located in the center of the system, with a 0.25 cm projection width on the isocenter treatment plane, while the remaining 28 half‐leaf pairs with 0.5 cm projection width are located on the outside.

### 2.2 Deviation of MLC position

The log files “dynalog”^16^ is used in the Varian Linear Accelerator to record mechanical information during treatment. Every 0.05 s, it reads and saves the planning parameters for the treatment plans and actual delivery, including the mechanical information of the gantry, collimator, jaws and leaves positions, which can be used to verify delivery accuracy after the treatment has completed. The deviation of MLC position is defined as the leaves positional difference between treatment plan and the actual ones recorded in the “dynalog” files at each sample point, and deviation of the “dynalog” file is an average of all the sample points.

### 2.3 Calculation of integrated fluence and extraction of feature information

**FIGURE 1:**
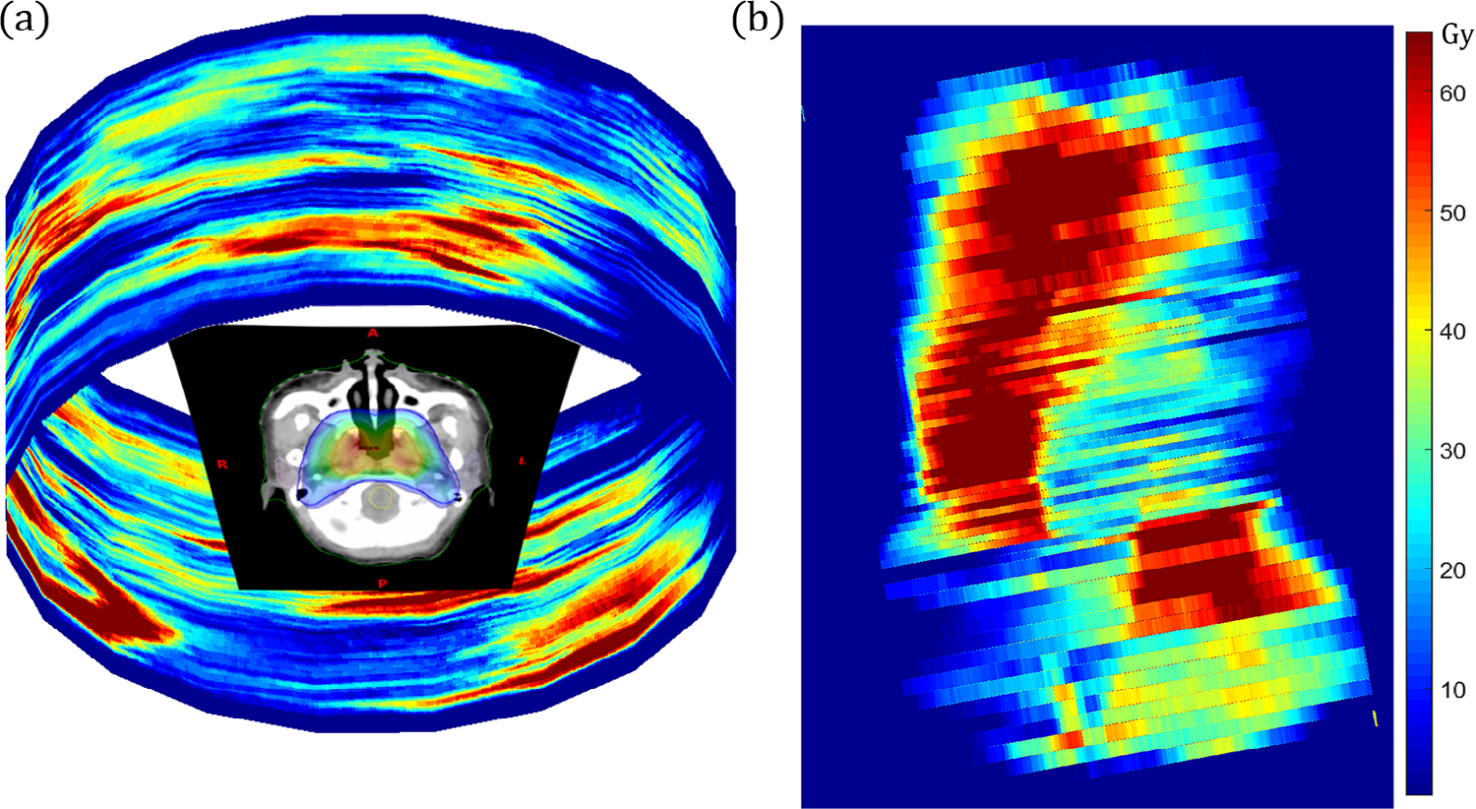
Fluence of VMAT plan. The actual delivery effect of VMAT (a) and integrated fluence of VMAT after edge enhancement (b) are shown.

Each CP's information is contained in the Dicom‐RT files of treatment plans. The shapes of the CPs and their corresponding MUs were read from these Dicom‐RT files using a self‐written Matlab function, and they were subsequently multiplied and superimposed to generate the integrated fluence (as shown in Figure 1b). During the actual treatment, the fluence of different CP will not be superimposed because they are delivered at different gantry angles (as shown in Figure 1a). However, since all the CPs were superimposed in the integration fluence, it may potentially cause the accumulation of several small CPs to be consistent with a single large CP, which could smear out the shapes of these small CPs. We have twice as much intensive augmentation applied to each CP edge's pixels in light of this (shown as the dark CP edges in Figure 1b). For the purpose of a realistic simulation of treatment plan, we retained the original angle of the collimator generated an integrated fluence of 1792*2388 pixels, which is determined by the size of the largest CP in the treatment plan.

These integrated fluences are used for the extraction of feature information. When analyzing spatial distribution characteristics, the gray level co‐occurrence matrices (GLCM) of the edge‐enhance integrated fluence were calculated, and textural features of correlation, contrast, energy, entropy and homogeneity were calculated, whose formulas are shown in Equations (1)–(5) below:

(1) $$correlation\;=\sum_{i=1}^{k}\sum_{j\;=\;1}^{k}\frac{\left(i-\;Mean\right)*\left(j-\;Mean\right)*p{\left(i,j\right)}^{2}}{\;Variance\;}$$

(2) $$contrast\;=\sum_{i=1}^{k}\sum_{j\;=\;1}^{k}p\left(i,j\right){\left(i-j\right)}^{2}$$

(3) $$energy=\sum_{i=1}^{k}\sum_{j\;=\;1}^{k}p{\left(i,j\right)}^{2}$$

(4) $$entropy\;=\;-\sum_{i\;=\;1}^{k}\sum_{j\;=\;1}^{k}p\left(i,j\right)\log p\left(i,j\right)$$

(5) $$homogeneity=\sum_{i=1}^{k}\sum_{j=1}^{k}\frac{1}{1+{\left(i-j\right)}^{2}}p\left(i,j\right)$$

where k is the pixel number in image p, and Mean and Variance are the image gray scale mean and variance, respectively.

**FIGURE 2:**
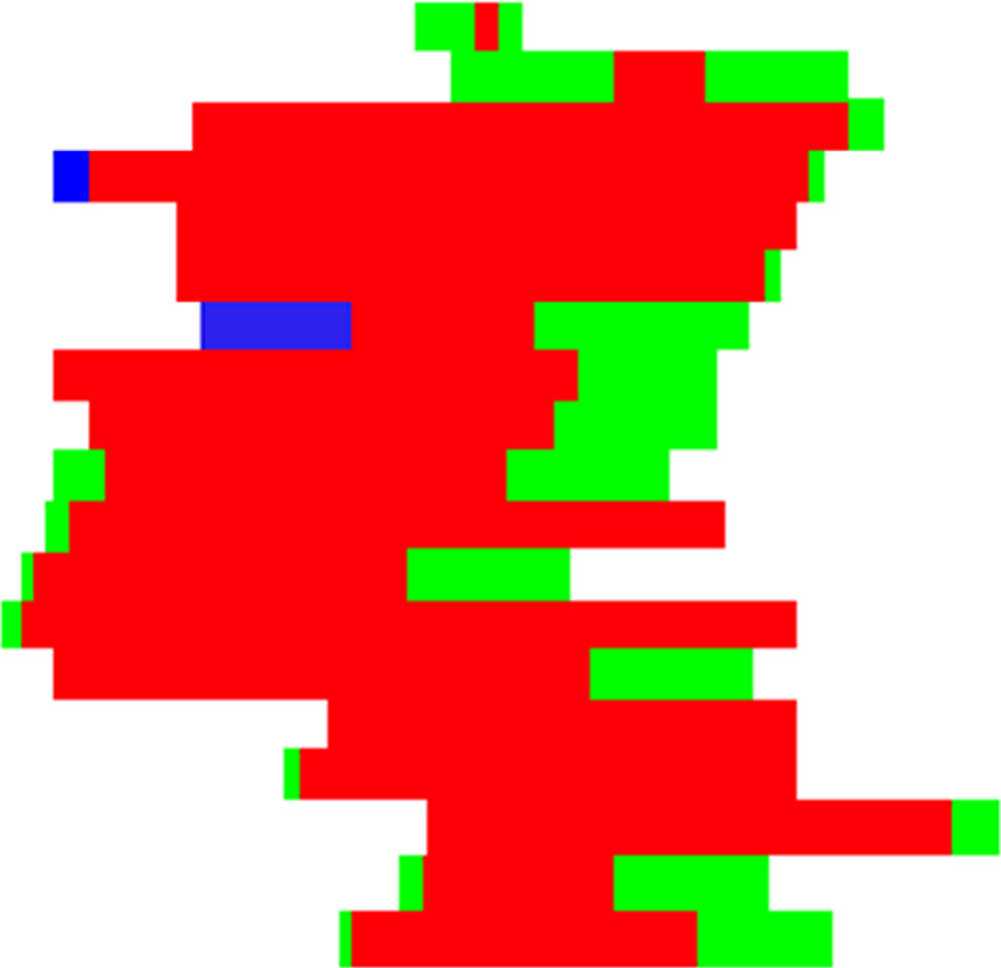
Integrated result of two adjacent CPs. The blue area and the green area are separate areas belonging to CP1 and CP2, respectively, while the red area is overlap.

On the other hand, we calculated the temporal variability by measuring the shape change of adjacent CPs, and the idea of Dice's Coefficient is used for reference. Figure 2 shows the integrated result of two adjacent CPs with changes in shape, where the blue area and the green area are separate areas belonging to CP1 and CP2, respectively, while the red area is overlap. The variability of these two CPs is defined as:

(6) $$Dice=\frac{Area_{overlap}\times \;2}{Area_{CP1}+Area_{CP2}}$$

where Dice is an indicator describing the variability. $Area_{CP1}$ and $Area_{CP2}$ represent the respective areas of the two adjacent CPs, while $Area_{overlap}$ is the area that they overlap. The Dice of one field is calculated from the average of all the variability of adjacent CPs contained in it. MATLAB (version R2016a) was used to read Dicom‐RT files for the treatment plan.

### 2.4 Mechanical wear parameters of MLC

Before using the Linac for treatment, a daily self‐check of MLC is performed on the management software called MLC‐hyper,^17^ and the performance parameters of each leaf will be recorded in a file with the suffix “init”, which was read using a self‐written code (programming on R (Version 4.0.2)) to evaluate the mechanical wear state of the MLC during treatment. The difference between the planning and actual positions of all MLC leaves for each plan were also obtained from the log files, whose average values were taken as the MLC deviations. note that since the MLC is equipped into A/B banks with different mechanical parameters, these MLC deviations need to be analyzed differently for these two banks as well. The potential mechanical wear parameters selected in this article are Maximum error(ME), backlash(BL), measured spring compression(MSC), pulse width modulation (PWM), leaf gaps (LG), leaf odometer(LO), and carriage odometer(CO).

Each of the VMAT plans for the 23 cases contains two fields and each field delivered generates two “dynalog” files. Only “dynalog” files with MLC mechanical wear parameters data of the same day were chosen for analysis. At least five treatments records were collected for each case, and a total of 744 “dynalog” files were obtained for modeling and analysis.

### 2.5 Multi‐correlation analysis

The multiple correlation coefficient is an index that measures the degree of linear correlation between a variable and other multiple variables.^18^ Assuming that there is a dependent variable y and independent variables x_1_, x_2_, …, x_k_, where the correlations between y and each variable in x_1_, x_2_, …, x_k_ cannot be directly measured. A linear combination about x_1_, x_2_, …, x_k_ can be constructed as:

(7) $$\hat{y}=\beta_{0}+\beta_{1}x_{1}+\beta_{2}x_{2}+\cdots +\beta_{k}x_{k}$$

where $\beta_{0}$, $\beta_{1}$, $\beta_{2}$ …, $\beta_{0}$ are the coefficients of linear combination. The correlation coefficient between y and the linear combination was calculated:

(8) $$R=\frac{\sum \left(y-\bar{y}\right)\left(\hat{y}-\bar{y}\right)}{\sqrt{\sum {\left(y-\bar{y}\right)}^{2}{\left(\hat{y}-\bar{y}\right)}^{2}}}$$

where $\bar{y}$ is the average of y, and R is defined as the multi‐correlation coefficient between variables y and x_1_, x_2_, …, x_k_.

The *p*‐value of the one‐sample Kolmogorov–Smirnov test for the data is less than 0.05, proving that this data does not conform to a normal distribution. To ensure the validity of the study, the Spearman's correlation coefficients(R) and corresponding *p*‐values of the MLC position deviation to the potential impact factors were first calculated to screen the effective ones. Secondly, the MLC positional deviation was used as the dependent variable, and the spatial characteristics and temporal variability of VMAT fluence together with the mechanical wear parameters of MLC were used as independent variables for multi‐correlation analysis. This operation was performed using the regression analysis function of SPSS(version 22).

### 2.6 Construction of prediction model

In this study, we introduced machine learning algorithm to fit all the influencing impacts with the deviation of MLC position and build a predictive model. The random forest (RF) obtains the prediction by aggregating classification or regression trees.^19^ Each tree of the RF is constructed using a different bootstrap sample of the data, and the best nodes are split among a subset of predictors randomly chosen.^20^ The Gini index is used as the splitting criterion.^21^ Considering that RF can be used for an identification of important associations among variables and perform both regression and classification analyses, and is especially suitable for the analysis of large datasets and identifying non‐linear relationships,^22^, ^23^, ^24^ it is selected as the main algorithm for predictive model construction in this research.

Our predictive model is designed as taking the effective spatial and temporal features of fluences (correlation, energy, entropy, homogeneity, and Dice), and the effective mechanical wear parameters of MLC (ME, BL, SC, PWM, LO, and CO) as the input, while the deviation of MLC position is as the output for fitting. The data set was randomly divided into training and test data in the ratio of 0.7:0.3, and the training data's mean, standard deviation, and maximum and minimum values were then used to standardize and normalize the data. To prevent the model from overfitting and to avoid random errors, a 5‐fold cross‐validation method was used during the model's training. The parameters to be set for RF include the number of nodes and decision trees, where the nodes are related to the number of inputs and the trees are chosen according to the fitting process. The fitting error tends to stabilize after the number of nodes and trees increase to a certain value, while too many nodes and trees leads to an increase of computation amount and a decrease of model accuracy. Both of these two numbers need to be determined experimentally in the training data. The model prediction accuracy is evaluated in terms of Mean Squared Error (MSE) and RF own var explanatory degree:

(9) $$MSE=\frac{1}{m}\sum_{i\;=\;1}^{m}{\left(y_{i}-{\hat{y}}_{i}\right)}^{2}$$

where m is the number of test samples, while $\hat{y}$ and y are the predicted value and the actual value, respectively. In order to compare and verify the effect of the RF model, the fitting result of linear model (LM) is also presented, which is one of the simplest forms, well interpreted, and commonly used fitting methods. The RF model construction was implemented using the “randomForest” package of R (Version 4.0.2), while the LM model was constructed using the “lm()” function in R (Version 4.0.2).

### 2.7 Correlation analysis between MLC positional deviation and delivery quality

In order to evaluate the effect of the positional accuracy of MLC on dosage, “dynalog” files of all the case executed on the same day as QA are collected for analysis. The actuary MLC leaf positions recorded in “dynalog” files were obtained and imported into the TPS in Dicom‐RT format to recalculate the actual dose, and the Dicom‐RT writing was implemented on MATLAB (version R2016a). The average dose deviations of targets (PGTV/PTV1/PTV2) and part of the organ at risk (OAR) (BrainStem/SpinalCord/Parotid) of all cases are calculated.

An additional metric for assessing delivery accuracy is the gamma passing rate. Dose distribution in a 24.4 cm * 24.4 cm plane was measured with a 32 * 32 detector array of MatriXX (myQA‐SQL_TN002, IBA Dosimetry GmbH, Germany) installed into the MULTICube (myQA‐SQL_TN002, IBA Dosimetry GmbH, Germany). The distance between each detector center is 0.762 cm, that is, the measurement is sampled at 1.78 detectors per cm^2^. These measured dose distributions were then compared to the 2D planar dose distributions calculated in TPS with a 0.25 mm grid size. Prior to measurements, MatriXX's sensitivity and absolute dosage calibrations were completed. In addition to the 3%/2 mm criteria suggested by task group (TG) 218 report of the American Association of Physicists in Medicine (AAPM),^25^ the global gamma passing rates of 2%/2 mm was also obtained for analysis.

A correlation analysis was conducted to determine the effect of MLC positional accuracy on planning delivery quality, which involved calculating the Spearman's correlation coefficients (R) and accompanying *p*‐values (with a value less than 0.05 was considered statistically significant) for positional deviation, dosage deviations, and gamma passing rates. It evaluates the monotonous relationships between continuous or sequential variables and does not require these variables to be normally distributed. R (version 4.0.2) and SPSS (version 22) were used for statistical analysis and plotting of data.

## 3 RESULTS

### 3.1 Deviation of MLC position

**FIGURE 3:**
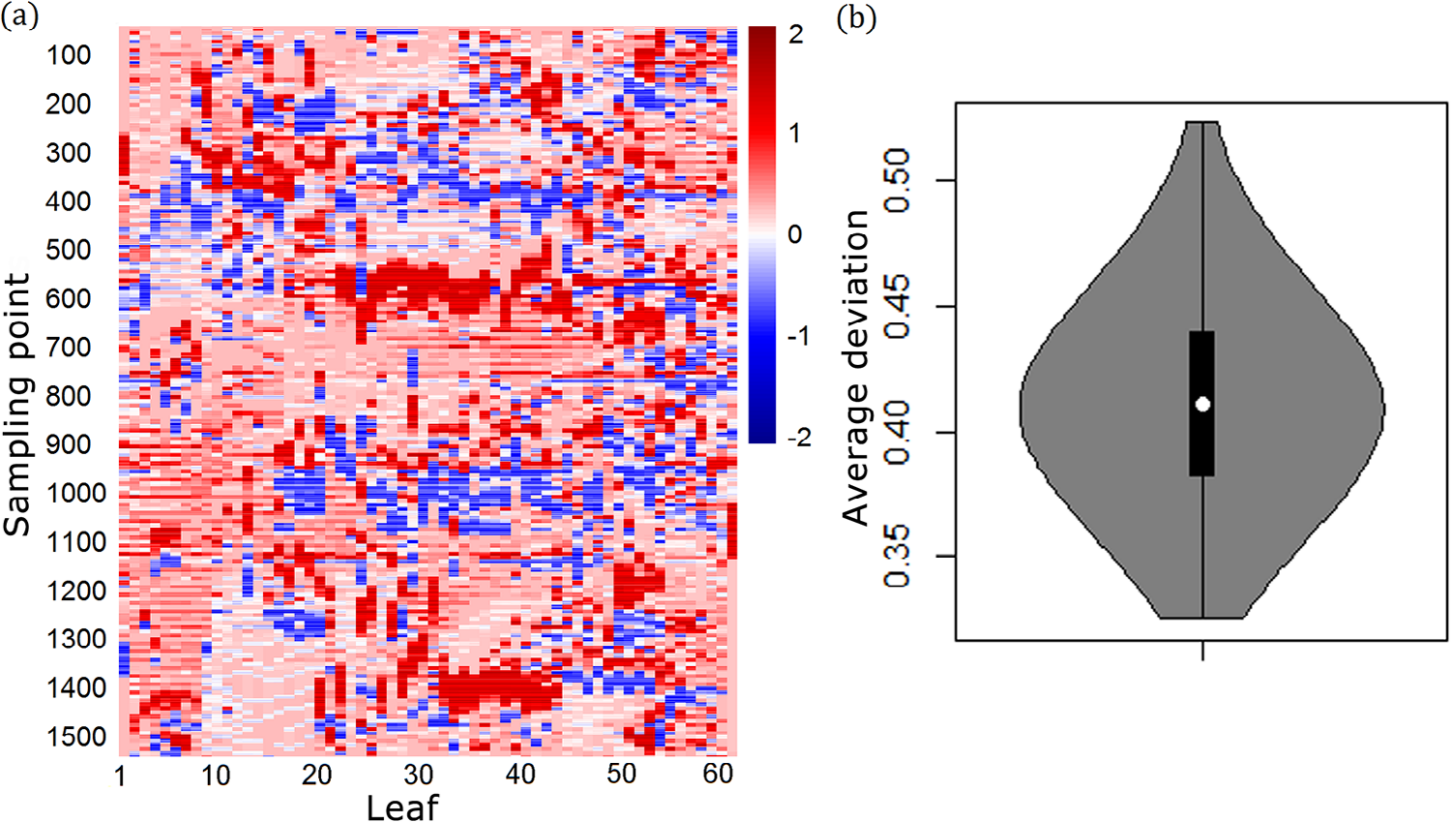
MLC positional deviation. A heatmap of MLC positional deviation for bank A of one field (a) and the average deviation distribution of 744 “dynalog” files in the form of violin‐box plot (b) are shown. The box plot in (b) shows the quantile of the average deviations, while the kernel density shows the density of the average deviation at the corresponding position.

The actual delivery of the treatment plan is recorded in the “dynalog” files according to the fields and A/B banks. A heatmap of MLC positional deviation is shown in Figure 3(a) (only for bank A of one field). The field contains a total of 1514 sampling points (distinguished by row, sampling every 0.05 s), and the deviations of 60 leaves (distinguished by column) is defined as the actual position value minus the expected one. The average absolute deviation of this “dynalog” file is 0.38 mm, of which the sampling points with absolute deviation greater than 1 mm accounted for 9.9%, while the maximum absolute deviation value can reach 2.21 mm.

Similarly, by averaging the deviations of all sampling points, the average positional accuracy of each “dynalog” file can be obtained. What should be noted is that the leaves are in reciprocating motion during the execution of VMAT plan, which would result in a greater or less comparison result between the actual position value and the planned one in each sampling point. Considering that the deviation values ​​with positive and negative signs counted will cause the offset of each other during accumulation, these deviation values ​​are taken as absolute values ​​before analysis. Figure 3(b) shows an average deviation distribution of 744 “dynalog” files in the form of violin‐box plot. It can be seen that there are deviations in all the “dynalog” files, and the distribution range of the average deviations are 0.32–0.52 mm, with an overall average of 0.41 mm. Files with deviation values near the average own the largest number.

### 3.2 Fluence feature extraction

**FIGURE 4:**
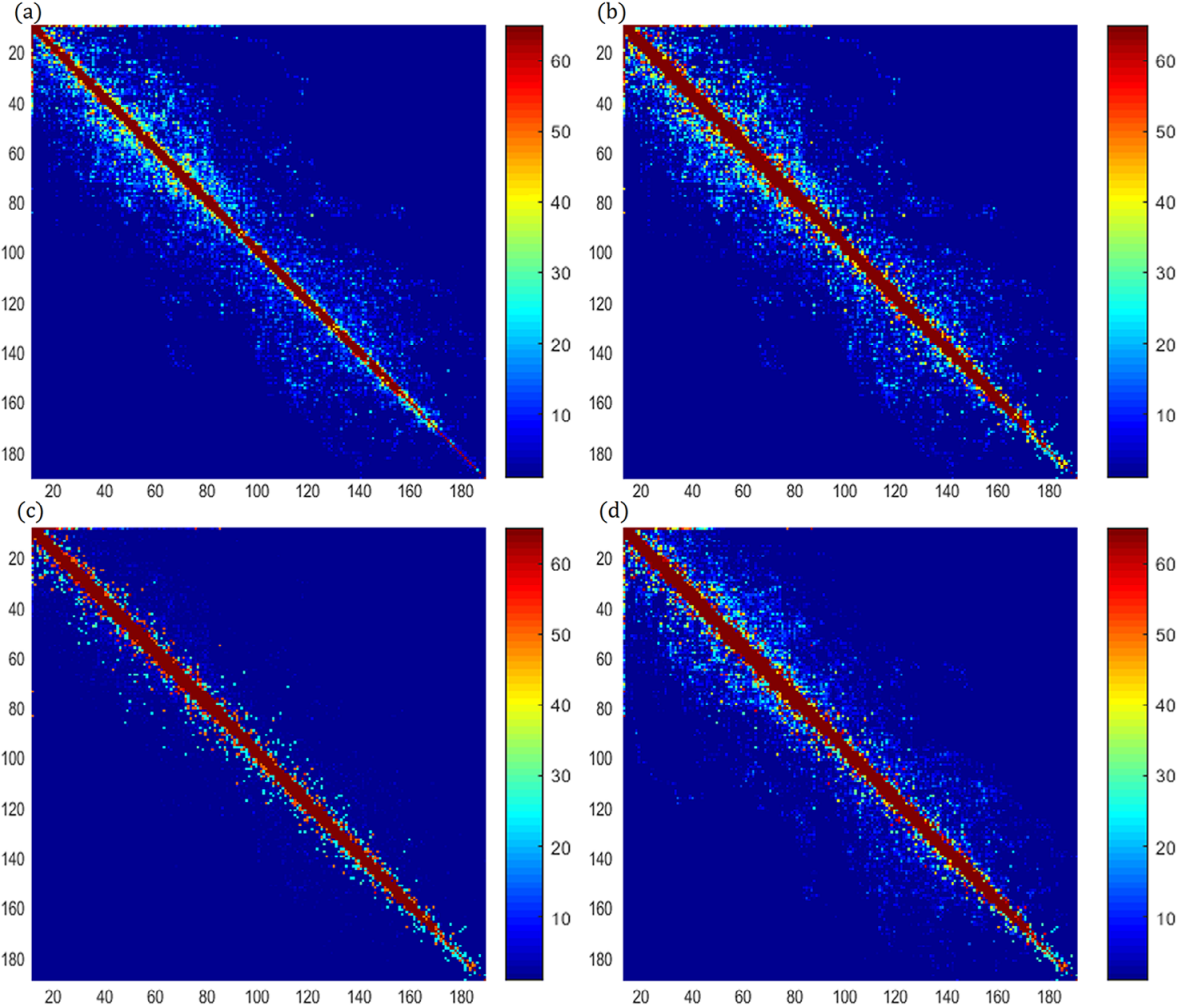
GLCM of one fluence in four directions. (a)–(d) are GLCM generated by search angles of 0°, 45°, 90°, and 135°, respectively with a distance *d* = 1.

In the GLCM parameters setting, searching directions angles of 0°, 45°, 90°, and 135° with a distance of d = 1 pixel were set. Figure 4 shows the GLCM of one fluence in four directions. Textural features (correlation, contrast, energy, entropy, and homogeneity) were calculated from the GLCM, which will serve as the representation of spatial distribution characteristics of the integrated fluence. In addition, Dice index of the field reflects the temporal variability, which can be obtained by averaging all the dice of adjacent CPs according to Equation (1). The above feature values extracted from a total of 46 fields in 23 cases are shown in Table 1.

**TABLE 1:** The values of features calculated from fluences.

Features	Values
Spatial features	
Contrast	42.034 ± 8.172
Correlation	0.000376 ± 0.000076
Energy	0.186 ± 0.062
Entropy	4.421 ± 0.427
Homogeneity	0.878 ± 0.012
Temporal feature	
Dice	0.899 ± 0.013

### 3.3 Mechanical wear parameters

**FIGURE 5:**
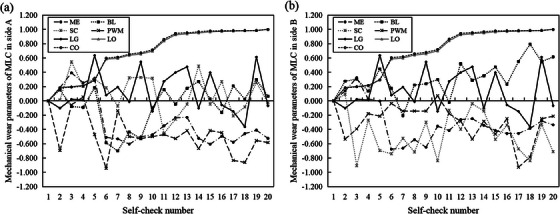
Normalized values of mechanical wear parameters of MLC calculated from init files. (a, b) show the wear parameters of the side A and side B, respectively, where the Self‐check number is used as the horizontal coordinate and the normalized value of the wear parameters is used as the vertical coordinate.

The treatment time span of the selected cases is from 2020.07 to 2020.10, with a total of 20 treatment records (files with the “init” suffix) were collected. Leaves parameters are averaged after distinguish by A/B banks, and the normalized values (normalized to the corresponding first value, respectively) of mechanical wear parameters of each treatment is obtained and shown as Figure 5. It can be seen that the A/B banks own different wear state each time, and with the number of treatments increasing, part of the parameters (BL, SC, LO, CO) show a slight upward trend.

### 3.4 Correlation analysis of MLC positional deviation to the potential impact factors

The Spearman correlation analysis results of MLC position deviation to all potential impact factors are shown in Table 2. It can be seen that except for the weak correlation of LG and Contrast (R = −0.021 and 0.020, with *p*‐value = 0.561 and 0.582, respectively), the remaining factors are all effective ones.

**TABLE 2:** Correlation analysis of MLC positional deviation to the potential impact factors.

	R	*p*‐value
Mechanical wear parameters		
ME	−0.411	<0.001
BL	0.425	<0.001
SC	0.414	<0.001
PWM	−0.439	<0.001
LG	−0.006	0.872
LO	0.426	<0.001
CO	−0.348	<0.001
Spatial characteristics		
Correlation	−0.338	<0.001
Energy	−0.178	<0.001
Entropy	0.272	<0.001
Contrast	0.030	0.421
Homogeneity	−0.362	<0.001
Temporal variability		
Dice	−0.320	<0.001

Abbreviation: R, Spearman's correlation coefficient.

In the multi‐correlation analysis, the MLC positional deviation was used as the dependent variable, and all the effective factors (correlation, energy, entropy, homogeneity, dice, ME, BL, SC, PWM, LO, and CO) were used as independent variables. A multi‐correlation coefficient of R = 0.701 with *p*‐value < 0.05 is obtained, which means that there is a strong correlation between the selected factors to MLC positional deviation.^26^

### 3.5 RF predictive model construction and result evaluation

**FIGURE 6:**
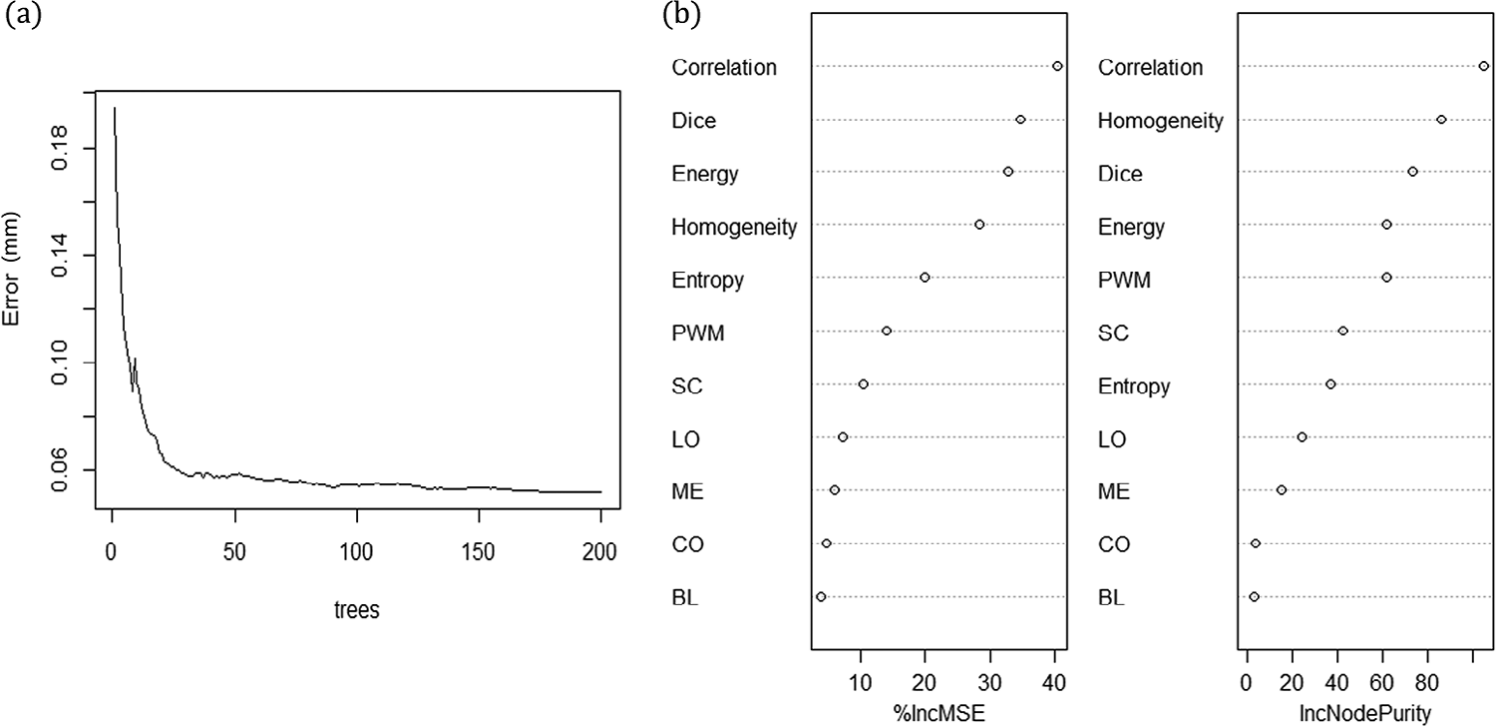
The predictive model of RF. The fitting process (a) and contribution statistics of input factors (b) of the RF model are shown.

There are 521 and 223 “dynalog” files in the training data and test data, respectively. The parameters of RF model are determined as node's number of 6 and tree's number of 200 after repeated tests. Fitting process for one of the folds of the RF model is shown in Figure 6(a), which presents the relationship between the model error (i.e., mean of squared residuals) and the tree's number during model training for the training data. It demonstrated high predictive success for the training data with average variables explained of 95.03% and average MSE of 0.059 in the 5‐fold cross‐validation, and a MSE of 0.074 for the test data was obtained.

Contribution statistics of each input factor in the RF model are also calculated and shown as Figure 6(b). It can be seen that the homogeneity and correlation of the spatial features, and the dice of the temporal feature account for the highest contribution value, while PWM and Energy are the second. Other factors of mechanical wear parameters (LO, ME, CO, and BL) are ranked behind.

**FIGURE 7:**
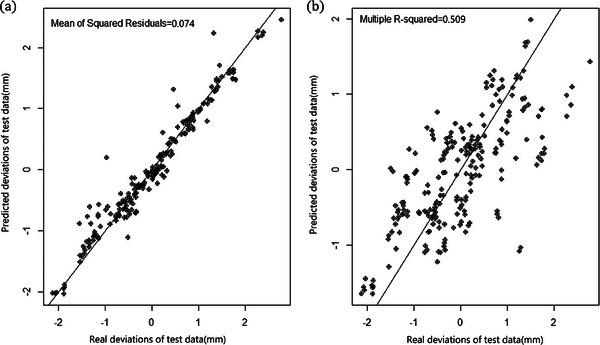
Comparison results of the test data in RF (a) and LM (b) for real deviations versus predicted deviations.

In order to compare the prediction effects of the RF model, the same training data and test data are also fitted with LM, and a MSE of 0.509 for the test data was obtained. The comparison results of the test data in these two models for real deviations versus predicted deviations were shown in Figure 7, which reflects that the RF model shows a better performance.

### 3.6 Dose deviation statistics affected by MLC positional deviation

A total of 92 “dynalog” files of all QA cases recorded on the same day were collected, and the actual MLC position of these files were obtained and imported into the TPS for dosage recalculation. The dose deviation statistics for targets and part of the OARs are shown in Table 3. There are four dynalog files (two fields with A/B banks) contains in the treatment record of one case, and the MLC deviation of the treatment is calculated from the average deviation of all these four dynalog files. The global gamma passing rates of QA plans with criteria of 2%/2 mm and 3%/2 mm were acquired, and Spearman's correlation coefficients (R) and corresponding *p*‐values of positional deviation to dose deviations and gamma passing rates were calculated.

**TABLE 3:** Correlation analysis of MLC positional deviation to dose deviations and gamma passing rates.

	M ± SD	R	*p*‐value
MLC positional deviations (mm)	0.412 ± 0.027	*–*	*–*
Dose deviations			
Dmax of PGTV (cGy)	32.978 ± 26.481	0.485	0.019
Coverage of PGTV (%)	2.494 ± 1.771	−0.084	0.705
Coverage of PTV1 (%)	0.470 ± 0.196	0.046	0.835
Coverage of PTV2 (%)	1.725 ± 0.281	0.023	0.916
Dmax of BrainStem (cGy)	56.717 ± 43.62	0.038	0.863
Dmean of BrainStem (cGy)	28.491 ± 16.311	−0.226	0.299
Dmax of SpinalCord (cGy)	26.448 ± 16.165	0.184	0.400
Dmean of SpinalCord (cGy)	12.948 ± 9.59	0.158	0.472
V30 of Parotid‐L (%)	5.161 ± 1.384	−0.397	0.061
Dmean of Parotid‐L (cGy)	210.235 ± 45.182	−0.367	0.085
V30 of Parotid‐R (%)	2.345 ± 1.400	−0.126	0.568
Dmean of Parotid‐R (cGy)	89.396 ± 38.726	−0.018	0.934
Gamma passing rates			
2%/2 mm (%)	92.552 ± 4.248	−0.656	<0.001
3%/2 mm (%)	97.148 ± 2.521	−0.518	0.011

Abbreviation: M, mean value; R, Spearman's correlation coefficient; SD, standard deviation.

Results show that the absolute dose deviation can range from 12.948 to 210.235 cGy, while the relative volume deviation is small (0.470%–5.161%). However, in the correlation analysis to MLC positional deviation, in addition to the weakly correlation of Dmax of PGTV, V30 of Parotid‐L, Dmean of Parotid‐L (0.485, −0.397 and −0.367, respectively), there is no correlation among other quantities (correlation value < 0.3). On the other hand, MLC positional deviation and passing rate of gamma show a strong correlation (2%/2 mm and 3%/2 mm are −0.656 and −0.518, respectively, with *p* < 0.05).

## 4 DISCUSSION

According to the RF model's input factor contribution statistics, texture and fluence variability are the primary effects that account for the largest contribution value. The results of the studies^13^, ^14^ show that for MLC positional errors, the strong correlation was found between Correlation (d = 1) and MLC errors (r_s_ = −0.766 with *p* < 0.001), which is consistent with our study. However, mechanical wear (PWM) continues to be a significant concern that cannot be disregarded. Each leaf of the standard MLC is driven by a separate motor, and the rotary motion is converted into a linear motion by a nut and a screw, which is defined as a mechanical motion unit.^15^ The motion unit is aging due to extended clinical use, which can cause leaves to take longer to achieve their intended position and may even result in errors when position deviations surpass threshold values. As a result, mechanical wear has a detrimental effect on MLC positioning accuracy that requires attention. In fact, as the wear and tear parts of motion unit, regular maintenance and replacement for the motors and nuts is necessary.

The modeling reveals the contribution of different mechanical wear parameters to MLC deviations. Therefore, the first application is to suggest the mechanical parameters (e.g., PWM) that should be paid more attention to during routine MLC maintenance. Secondly, this study found a strong correlation between MLC average positional deviation and gamma passing rate, and a close relationship between plan pass rates and their complexity has already been reported^27^, ^28^ so the second potential application of this study is to inform the control of plan complexity. Overly complex plans may affect the MLC deviations during actual plan delivery in addition to the decrease of the passing rate, and a planning complexity threshold setting may be used to avoid a worse‐case scenario. What should be note is that the input‐output data of the model are acquired in the same day. Therefore, it is necessary to obtain the day's wear parameters in the model application to estimate the day's MLC positional deviation. In addition, MLC repairs occurred during the data collection of this study, and the selected MLC mechanical wear parameters data included records after these repairs, which were presented as mutation points in the trend of the attrition parameters (as shown in Figure 5). That is, the model has taken the effect of MLC repairs into account.

Osman et al.^8^ takes leaf position, gantry angle, beam on/off status, etc. as inputs, with an artificial neural network (ANN) of 20‐3‐1 hidden layers and neurons was used to predict the positional deviation of individual multi‐leaf collimator in each sampling point, and an accuracy with MSE having a value of 0.0001mm^2^ (RMSE = 0.0097 mm) was assessed. However, the results of our experiments show that weak correlation was found between these factors and the positional deviation of individual leaf (with correlation value < 0.1), and the results cannot be reproduced by the same methods in our experiments. The difference of MLC type may be one of the reasons for this result. Carlson et al.^3^ calculated leaf motion parameters from DICOM‐RT files such as leaf position, velocity, acceleration and motion direction, to predict leaves positions for moving and resting. The model based on the cubist algorithm outperformed all other different models (MAE = 0.253–0.278 mm, with RMSE = 0.371–0.426 mm). Which, in terms of magnitude, concurs with the outcome of our study. As far as we are concerned, there will be numerous factors influencing each leaf's accuracy, which will lead to a large contingency of the deviation result and lower statistical significance. Because of this, we take the average accuracy of all CPs in each one “dynalog” file as the research object.

In order to predict the patient‐specific QA (gamma passing rate), some recently published studies have investigated different models such as Poisson regression with Lasso regularization,^29^, ^30^ convolutional neural networks(CNN),^31^, ^32^ decision‐tree,^33^ RF,^34^ and support vector machine models.^35^ In our study, we also tested an ANN model with two hidden layers and twenty and seven neurons, respectively. The MSE value of 0.140 indicates that this model performed no better than RF (0.074). We believe that because deep learning models (like CNN and ANN) have complicated network structures and too many parameters, they are prone to overfitting or non‐convergence when processing the simple nonlinear data of MLC accuracy. Therefore, the accuracy of these models is not always better than that of traditional ensemble learning models. On the other hand, correlation between MLC deviation and all the potential impact factors with each other was calculated, and non‐independent correlations between the impact factors was found. We have tested the use of principal component analysis (PCA) for data compression and dimensionality reduction before RF model construction and obtained slightly worse results (variables explained = 92.01%, MSE = 0.083). And modeling directly with the original impact factors was finally chosen from the aspect of modeling steps simplicity and information preservation.

Although the feasibility of using “dynalog” files for planning QA has been reported,^36^, ^37^, ^38^ we are more interested in whether MLC positional precision has an effect on organ‐specific dose deviation. The actual MLC location of the “dynalog” file and imported into TPS to recalculate the dose, and the dose deviation statistics for targets and part of the OARs were analyzed. It is noteworthy that the dose statistics data indicate a slight correlation—which is to be expected—between dosage deviation and MLC positional deviation. Rather than the average deviation of all leaves, the dose of the target or OAR is actually influenced by individual or several leaves that correspond to its position in the CP. Gamma passing rate, on the other hand, calculates the variation in the global dose distribution between the intended and actual doses. The positional accuracy of each leaf in the field will affect its results. This explains also why the gamma passing rate has a strong correlation with the average positional deviation of MLC (especially the result of 2%/2 mm criteria that enlarged the gap of passing rates), but shows weak correlation with the actual dose deviations.^39^, ^40^, ^41^ In order to predict the dose of specific organs, it is still necessary to refine the accuracy analysis of MLC position to corresponding leaves, which will be our future work.

There are some limitations of this study. Firstly, the VMAT plans chosen for model construction were all NPC cases, the planning and delivery of which were performed on specific TPS and linear accelerator, respectively. When the model is used for the evaluation of other diseases, or there are changes in radiotherapy equipment, the cases data need to be updated for model reconstruction. Secondly, there is small number of VMAT plans included in the study, and we will add and screen more high‐quality case data at a later stage, and propose to use deep learning method to construct a more accurate model.

## 5 CONCLUSIONS

To study the factors that may affect the accuracy of MLC position, the spatial characteristics, temporal variability of fluence and comprehensive mechanical wear of MLC were extracted. A RF prediction model was fitted to all the effective factors and the MLC position deviation; this resulted in a high predictive accuracy with variables explained of 95.03% and MSE of 0.074. In addition, correlation between MLC positional deviation, dose deviations and gamma passing rates were analyzed to estimate the impact of MLC positional accuracy on planning delivery quality. The average positional deviation of the MLC has a limited link with dosage deviations, but a substantial correlation with gamma passing rates, according to the results. According to the study, the RF predictive model can be used as a priori tool for the accuracy prediction of MLC position.

## AUTHOR CONTRIBUTIONS

These authors contributed equally to this work.

## CONFLICT OF INTEREST STATEMENT

The authors have no relevant conflicts of interest to disclose.
